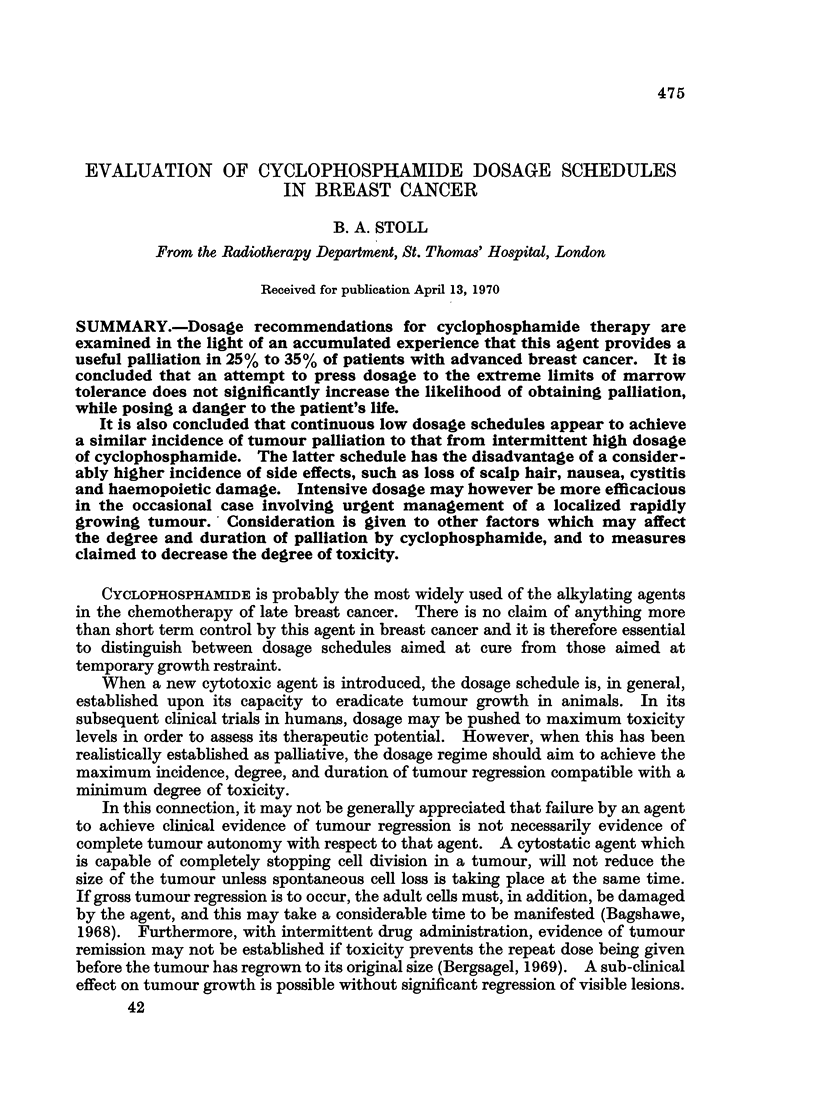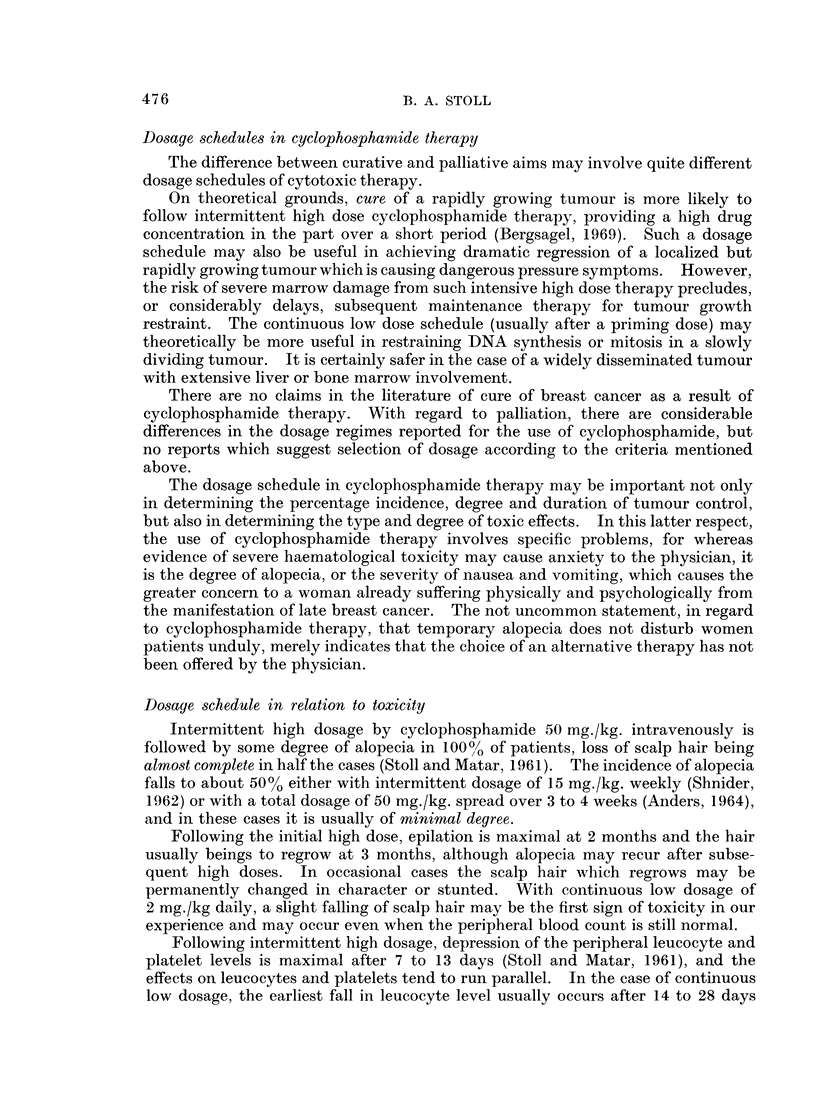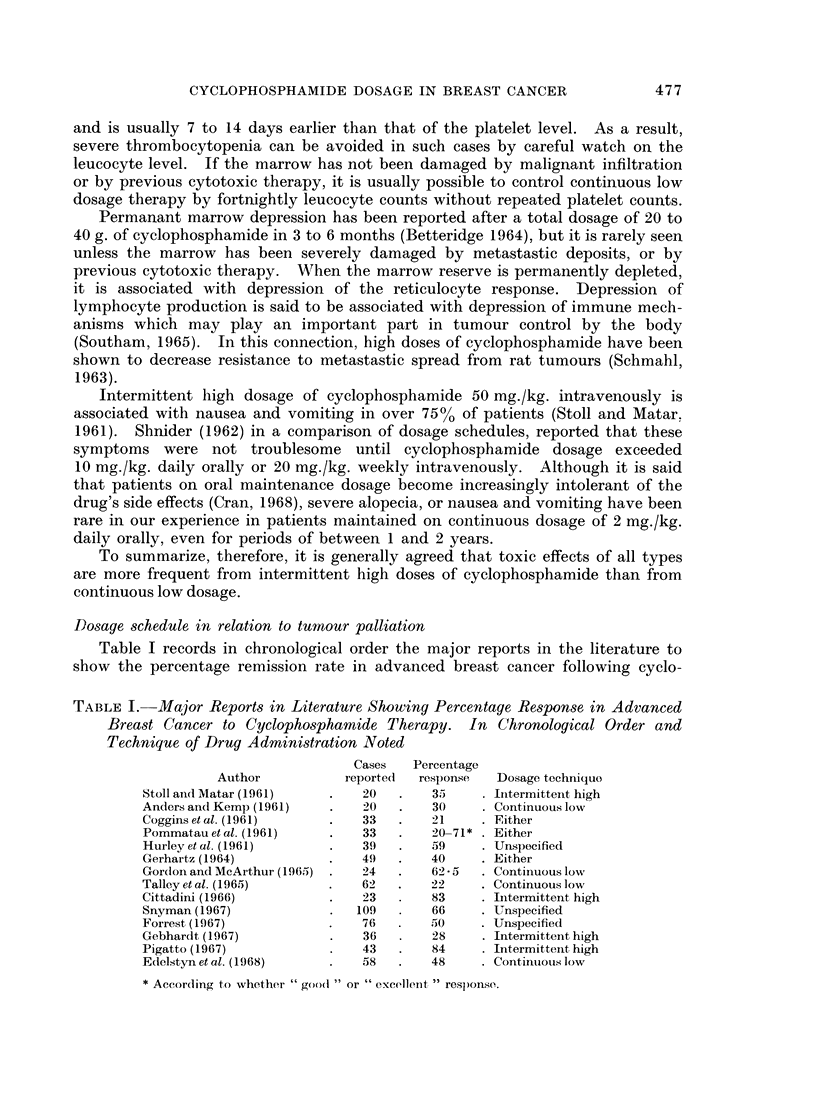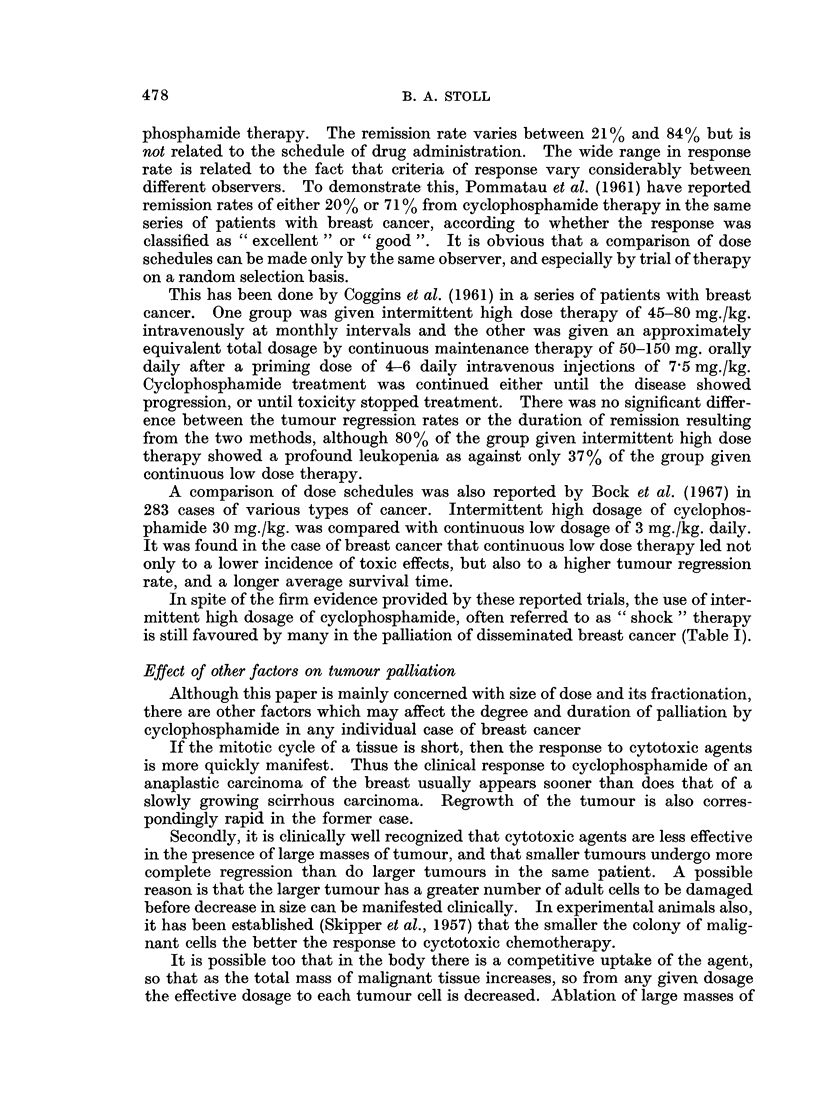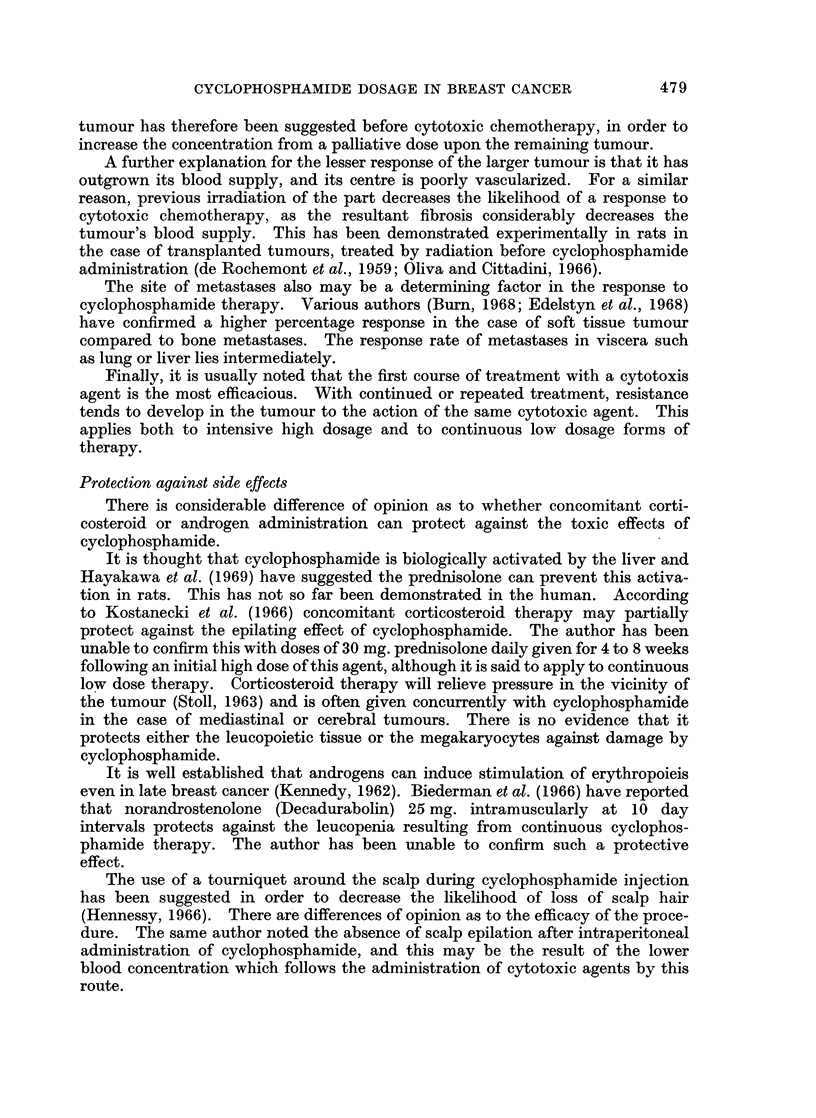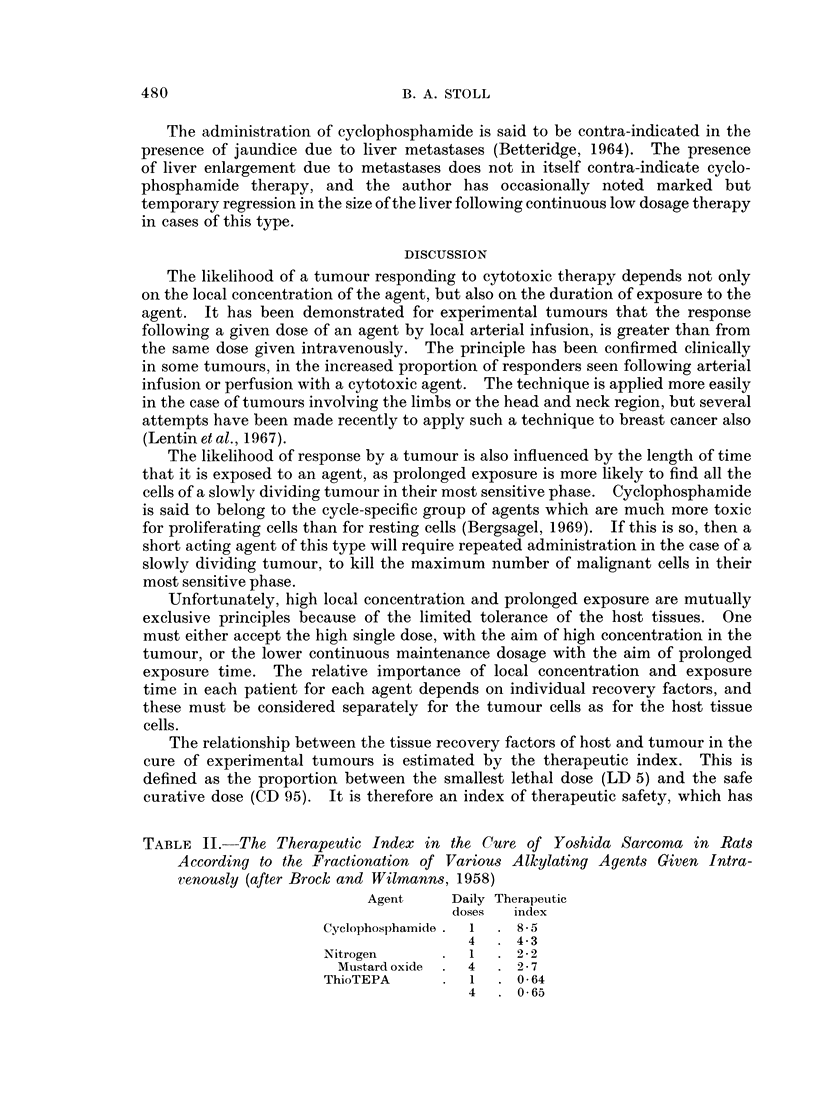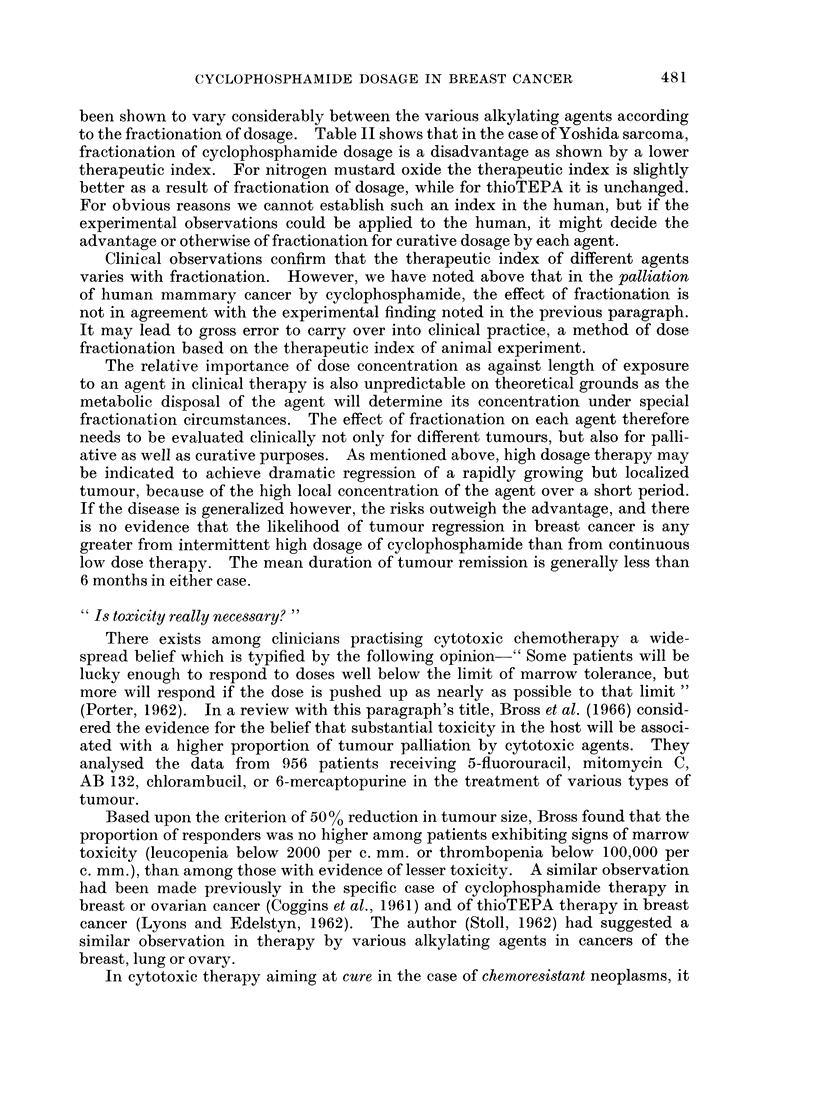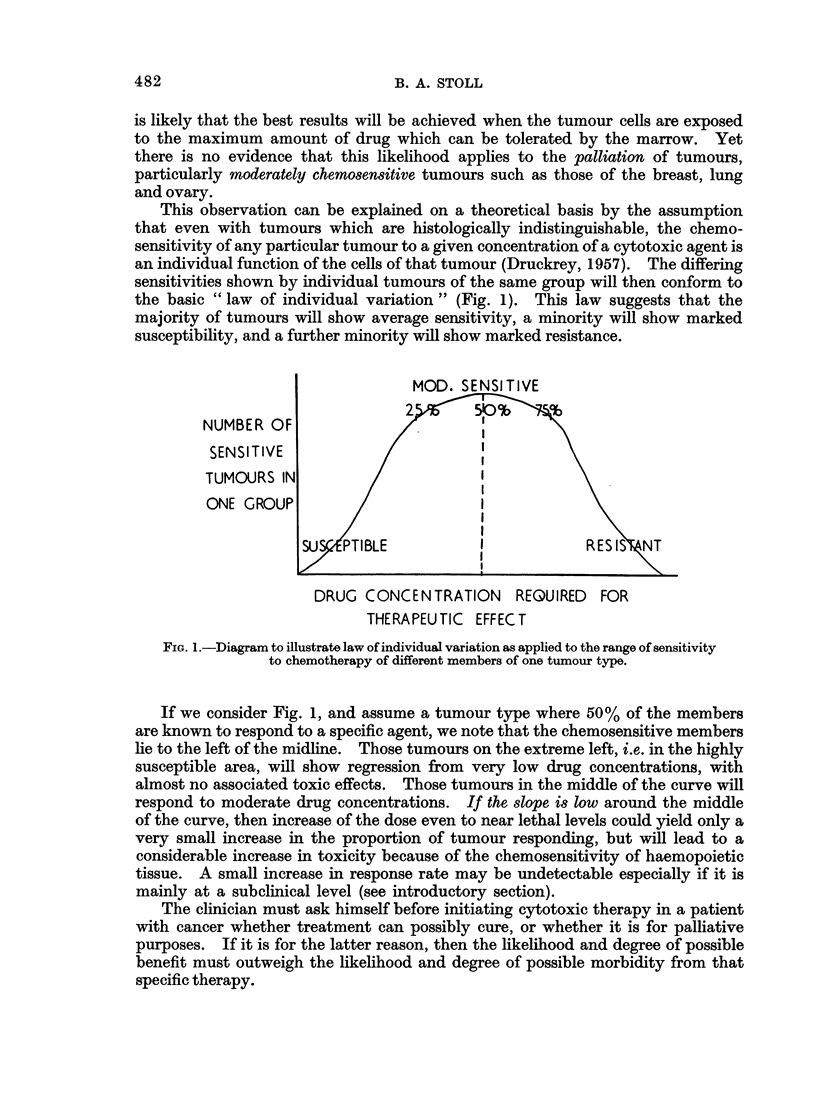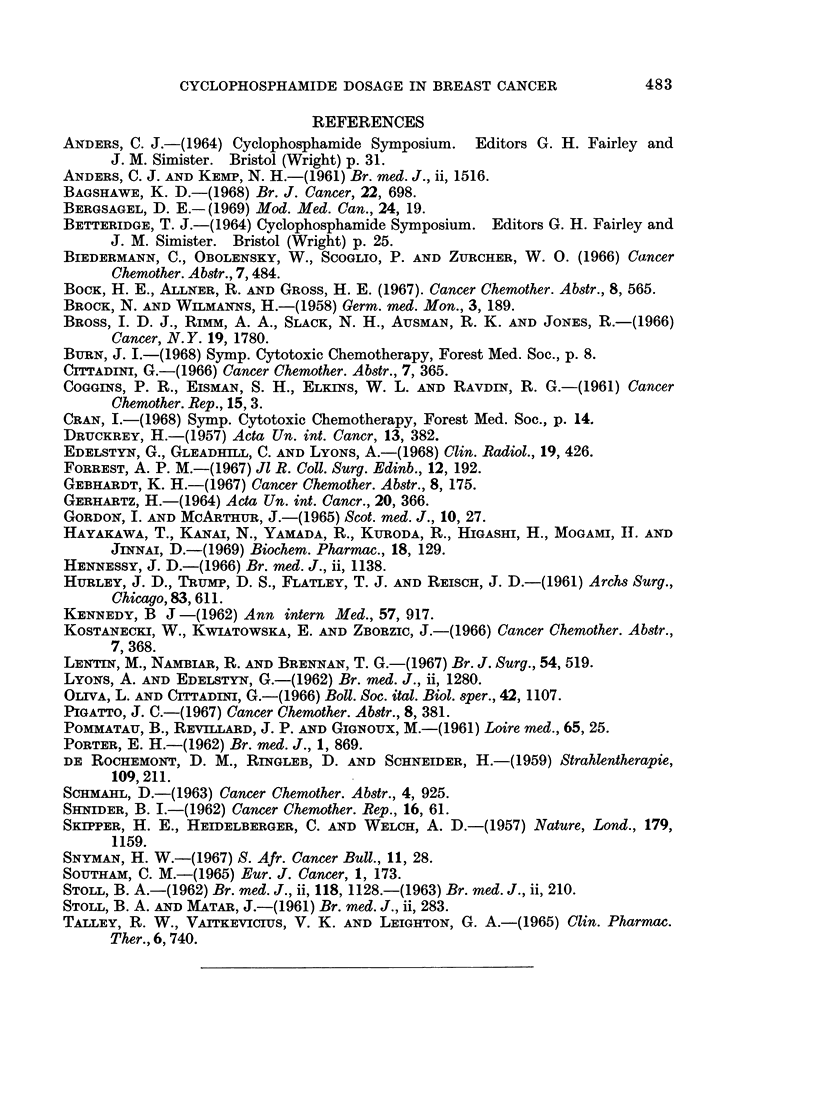# Evaluation of Cyclophosphamide Dosage Schedules in Breast Cancer

**DOI:** 10.1038/bjc.1970.57

**Published:** 1970-09

**Authors:** B. A. Stoll

## Abstract

Dosage recommendations for cyclophosphamide therapy are examined in the light of an accumulated experience that this agent provides a useful palliation in 25% to 35% of patients with advanced breast cancer. It is concluded that an attempt to press dosage to the extreme limits of marrow tolerance does not significantly increase the likelihood of obtaining palliation, while posing a danger to the patient's life.

It is also concluded that continuous low dosage schedules appear to achieve a similar incidence of tumour palliation to that from intermittent high dosage of cyclophosphamide. The latter schedule has the disadvantage of a considerably higher incidence of side effects, such as loss of scalp hair, nausea, cystitis and haemopoietic damage. Intensive dosage may however be more efficacious in the occasional case involving urgent management of a localized rapidly growing tumour. Consideration is given to other factors which may affect the degree and duration of palliation by cyclophosphamide, and to measures claimed to decrease the degree of toxicity.


### EVALUATION OF CYCLOPHOSPHAMIDE DOSAGE SCHEDULES IN BREAST CANCER

B. A. STOLL

*From the Radiotherapy Department, St. Thomas' Hospital, London*

Received for publication April 13, 1970

**SUMMARY.—Dosage recommendations for cyclophosphamide therapy are examined in the light of an accumulated experience that this agent provides a useful palliation in 25% to 35% of patients with advanced breast cancer. It is concluded that an attempt to press dosage to the extreme limits of marrow tolerance does not significantly increase the likelihood of obtaining palliation, while posing a danger to the patient's life.**

**It is also concluded that continuous low dosage schedules appear to achieve a similar incidence of tumour palliation to that from intermittent high dosage of cyclophosphamide. The latter schedule has the disadvantage of a considerably higher incidence of side effects, such as loss of scalp hair, nausea, cystitis and haemopoietic damage. Intensive dosage may however be more efficacious in the occasional case involving urgent management of a localized rapidly growing tumour. Consideration is given to other factors which may affect the degree and duration of palliation by cyclophosphamide, and to measures claimed to decrease the degree of toxicity.**

CYCLOPHOSPHAMIDE is probably the most widely used of the alkylating agents in the chemotherapy of late breast cancer. There is no claim of anything more than short term control by this agent in breast cancer and it is therefore essential to distinguish between dosage schedules aimed at cure from those aimed at temporary growth restraint.

When a new cytotoxic agent is introduced, the dosage schedule is, in general, established upon its capacity to eradicate tumour growth in animals. In its subsequent clinical trials in humans, dosage may be pushed to maximum toxicity levels in order to assess its therapeutic potential. However, when this has been realistically established as palliative, the dosage regime should aim to achieve the maximum incidence, degree, and duration of tumour regression compatible with a minimum degree of toxicity.

In this connection, it may not be generally appreciated that failure by an agent to achieve clinical evidence of tumour regression is not necessarily evidence of complete tumour autonomy with respect to that agent. A cytostatic agent which is capable of completely stopping cell division in a tumour, will not reduce the size of the tumour unless spontaneous cell loss is taking place at the same time. If gross tumour regression is to occur, the adult cells must, in addition, be damaged by the agent, and this may take a considerable time to be manifested (Bagshawe, 1968). Furthermore, with intermittent drug administration, evidence of tumour remission may not be established if toxicity prevents the repeat dose being given before the tumour has regrown to its original size (Bergsagel, 1969). A sub-clinical effect on tumour growth is possible without significant regression of visible lesions.

*Dosage schedules in cyclophosphamide therapy*

The difference between curative and palliative aims may involve quite different dosage schedules of cytotoxic therapy.

On theoretical grounds, *cure* of a rapidly growing tumour is more likely to follow intermittent high dose cyclophosphamide therapy, providing a high drug concentration in the part over a short period (Bergsagel, 1969). Such a dosage schedule may also be useful in achieving dramatic regression of a localized but rapidly growing tumour which is causing dangerous pressure symptoms. However, the risk of severe marrow damage from such intensive high dose therapy precludes, or considerably delays, subsequent maintenance therapy for tumour growth restraint. The continuous low dose schedule (usually after a priming dose) may theoretically be more useful in restraining DNA synthesis or mitosis in a slowly dividing tumour. It is certainly safer in the case of a widely disseminated tumour with extensive liver or bone marrow involvement.

There are no claims in the literature of cure of breast cancer as a result of cyclophosphamide therapy. With regard to palliation, there are considerable differences in the dosage regimes reported for the use of cyclophosphamide, but no reports which suggest selection of dosage according to the criteria mentioned above.

The dosage schedule in cyclophosphamide therapy may be important not only in determining the percentage incidence, degree and duration of tumour control, but also in determining the type and degree of toxic effects. In this latter respect, the use of cyclophosphamide therapy involves specific problems, for whereas evidence of severe haematological toxicity may cause anxiety to the physician, it is the degree of alopecia, or the severity of nausea and vomiting, which causes the greater concern to a woman already suffering physically and psychologically from the manifestation of late breast cancer. The not uncommon statement, in regard to cyclophosphamide therapy, that temporary alopecia does not disturb women patients unduly, merely indicates that the choice of an alternative therapy has not been offered by the physician.

*Dosage schedule in relation to toxicity*

Intermittent high dosage by cyclophosphamide 50 mg./kg. intravenously is followed by some degree of alopecia in 100% of patients, loss of scalp hair being *almost complete* in half the cases (Stoll and Matar, 1961). The incidence of alopecia falls to about 50% either with intermittent dosage of 15 mg./kg. weekly (Shnider, 1962) or with a total dosage of 50 mg./kg. spread over 3 to 4 weeks (Anders, 1964), and in these cases it is usually of *minimal degree.*

Following the initial high dose, epilation is maximal at 2 months and the hair usually beings to regrow at 3 months, although alopecia may recur after subsequent high doses. In occasional cases the scalp hair which regrows may be permanently changed in character or stunted. With continuous low dosage of 2 mg./kg daily, a slight falling of scalp hair may be the first sign of toxicity in our experience and may occur even when the peripheral blood count is still normal.

Following intermittent high dosage, depression of the peripheral leucocyte and platelet levels is maximal after 7 to 13 days (Stoll and Matar, 1961), and the effects on leucocytes and platelets tend to run parallel. In the case of continuous low dosage, the earliest fall in leucocyte level usually occurs after 14 to 28 days and is usually 7 to 14 days earlier than that of the platelet level. As a result, severe thrombocytopenia can be avoided in such cases by careful watch on the leucocyte level. If the marrow has not been damaged by malignant infiltration or by previous cytotoxic therapy, it is usually possible to control continuous low dosage therapy by fortnightly leucocyte counts without repeated platelet counts.

Permanant marrow depression has been reported after a total dosage of 20 to 40 g. of cyclophosphamide in 3 to 6 months (Betteridge 1964), but it is rarely seen unless the marrow has been severely damaged by metastastic deposits, or by previous cytotoxic therapy. When the marrow reserve is permanently depleted, it is associated with depression of the reticulocyte response. Depression of lymphocyte production is said to be associated with depression of immune mechanisms which may play an important part in tumour control by the body (Southam, 1965). In this connection, high doses of cyclophosphamide have been shown to decrease resistance to metastastic spread from rat tumours (Schmahl, 1963).

Intermittent high dosage of cyclophosphamide 50 mg./kg. intravenously is associated with nausea and vomiting in over 75% of patients (Stoll and Matar, 1961). Shnider (1962) in a comparison of dosage schedules, reported that these symptoms were not troublesome until cyclophosphamide dosage exceeded 10 mg./kg. daily orally or 20 mg./kg. weekly intravenously. Although it is said that patients on oral maintenance dosage become increasingly intolerant of the drug's side effects (Cran, 1968), severe alopecia, or nausea and vomiting have been rare in our experience in patients maintained on continuous dosage of 2 mg./kg. daily orally, even for periods of between 1 and 2 years.

To summarize, therefore, it is generally agreed that toxic effects of all types are more frequent from intermittent high doses of cyclophosphamide than from continuous low dosage.

*Dosage schedule in relation to tumour palliation*

Table I records in chronological order the major reports in the literature to show the percentage remission rate in advanced breast cancer following cyclo-

TABLE I.—*Major Reports in Literature Showing Percentage Response in Advanced Breast Cancer to Cyclophosphamide Therapy. In Chronological Order and Technique of Drug Administration Noted*

| Author | Cases reported | Percentage response | Dosage techniquo |
|---|---|---|---|
| Stoll and Matar (1961) | 20 | 35 | Intermittent high |
| Anders and Kemp (1961) | 20 | 30 | Continuous low |
| Coggins *et al.* (1961) | 33 | 21 | Either |
| Pommatau *et al.* (1961) | 33 | 20–71* | Either |
| Hurley *et al.* (1961) | 39 | 59 | Unspecified |
| Gerhartz (1964) | 49 | 40 | Either |
| Gordon and McArthur (1965) | 24 | 62·5 | Continuous low |
| Talley *et al.* (1965) | 62 | 22 | Continuous low |
| Cittadini (1966) | 23 | 83 | Intermittent high |
| Snyman (1967) | 109 | 66 | Unspecified |
| Forrest (1967) | 76 | 50 | Unspecified |
| Gebhardt (1967) | 36 | 28 | Intermittent high |
| Pigatto (1967) | 43 | 84 | Intermittent high |
| Edelstyn *et al.* (1968) | 58 | 48 | Continuous low |

* According to whether " good " or " excellent " response.

phosphamide therapy. The remission rate varies between 21% and 84% but is *not* related to the schedule of drug administration. The wide range in response rate is related to the fact that criteria of response vary considerably between different observers. To demonstrate this, Pommatau *et al.* (1961) have reported remission rates of either 20% or 71% from cyclophosphamide therapy in the same series of patients with breast cancer, according to whether the response was classified as " excellent " or " good ". It is obvious that a comparison of dose schedules can be made only by the same observer, and especially by trial of therapy on a random selection basis.

This has been done by Coggins *et al.* (1961) in a series of patients with breast cancer. One group was given intermittent high dose therapy of 45–80 mg./kg. intravenously at monthly intervals and the other was given an approximately equivalent total dosage by continuous maintenance therapy of 50–150 mg. orally daily after a priming dose of 4–6 daily intravenous injections of 7·5 mg./kg. Cyclophosphamide treatment was continued either until the disease showed progression, or until toxicity stopped treatment. There was no significant difference between the tumour regression rates or the duration of remission resulting from the two methods, although 80% of the group given intermittent high dose therapy showed a profound leukopenia as against only 37% of the group given continuous low dose therapy.

A comparison of dose schedules was also reported by Bock *et al.* (1967) in 283 cases of various types of cancer. Intermittent high dosage of cyclophosphamide 30 mg./kg. was compared with continuous low dosage of 3 mg./kg. daily. It was found in the case of breast cancer that continuous low dose therapy led not only to a lower incidence of toxic effects, but also to a higher tumour regression rate, and a longer average survival time.

In spite of the firm evidence provided by these reported trials, the use of intermittent high dosage of cyclophosphamide, often referred to as " shock " therapy is still favoured by many in the palliation of disseminated breast cancer (Table I).

*Effect of other factors on tumour palliation*

Although this paper is mainly concerned with size of dose and its fractionation, there are other factors which may affect the degree and duration of palliation by cyclophosphamide in any individual case of breast cancer

If the mitotic cycle of a tissue is short, then the response to cytotoxic agents is more quickly manifest. Thus the clinical response to cyclophosphamide of an anaplastic carcinoma of the breast usually appears sooner than does that of a slowly growing scirrhous carcinoma. Regrowth of the tumour is also correspondingly rapid in the former case.

Secondly, it is clinically well recognized that cytotoxic agents are less effective in the presence of large masses of tumour, and that smaller tumours undergo more complete regression than do larger tumours in the same patient. A possible reason is that the larger tumour has a greater number of adult cells to be damaged before decrease in size can be manifested clinically. In experimental animals also, it has been established (Skipper *et al.*, 1957) that the smaller the colony of malignant cells the better the response to cyctotoxic chemotherapy.

It is possible too that in the body there is a competitive uptake of the agent, so that as the total mass of malignant tissue increases, so from any given dosage the effective dosage to each tumour cell is decreased. Ablation of large masses of tumour has therefore been suggested before cytotoxic chemotherapy, in order to increase the concentration from a palliative dose upon the remaining tumour.

A further explanation for the lesser response of the larger tumour is that it has outgrown its blood supply, and its centre is poorly vascularized. For a similar reason, previous irradiation of the part decreases the likelihood of a response to cytotoxic chemotherapy, as the resultant fibrosis considerably decreases the tumour's blood supply. This has been demonstrated experimentally in rats in the case of transplanted tumours, treated by radiation before cyclophosphamide administration (de Rochemont *et al.*, 1959; Oliva and Cittadini, 1966).

The site of metastases also may be a determining factor in the response to cyclophosphamide therapy. Various authors (Burn, 1968; Edelstyn *et al.*, 1968) have confirmed a higher percentage response in the case of soft tissue tumour compared to bone metastases. The response rate of metastases in viscera such as lung or liver lies intermediately.

Finally, it is usually noted that the first course of treatment with a cytotoxis agent is the most efficacious. With continued or repeated treatment, resistance tends to develop in the tumour to the action of the same cytotoxic agent. This applies both to intensive high dosage and to continuous low dosage forms of therapy.

*Protection against side effects*

There is considerable difference of opinion as to whether concomitant corticosteroid or androgen administration can protect against the toxic effects of cyclophosphamide.

It is thought that cyclophosphamide is biologically activated by the liver and Hayakawa *et al.* (1969) have suggested the prednisolone can prevent this activation in rats. This has not so far been demonstrated in the human. According to Kostanecki *et al.* (1966) concomitant corticosteroid therapy may partially protect against the epilating effect of cyclophosphamide. The author has been unable to confirm this with doses of 30 mg. prednisolone daily given for 4 to 8 weeks following an initial high dose of this agent, although it is said to apply to continuous low dose therapy. Corticosteroid therapy will relieve pressure in the vicinity of the tumour (Stoll, 1963) and is often given concurrently with cyclophosphamide in the case of mediastinal or cerebral tumours. There is no evidence that it protects either the leucopoietic tissue or the megakaryocytes against damage by cyclophosphamide.

It is well established that androgens can induce stimulation of erythropoieis even in late breast cancer (Kennedy, 1962). Biederman *et al.* (1966) have reported that norandrostenolone (Decadurabolin) 25 mg. intramuscularly at 10 day intervals protects against the leucopenia resulting from continuous cyclophosphamide therapy. The author has been unable to confirm such a protective effect.

The use of a tourniquet around the scalp during cyclophosphamide injection has been suggested in order to decrease the likelihood of loss of scalp hair (Hennessy, 1966). There are differences of opinion as to the efficacy of the procedure. The same author noted the absence of scalp epilation after intraperitoneal administration of cyclophosphamide, and this may be the result of the lower blood concentration which follows the administration of cytotoxic agents by this route.

The administration of cyclophosphamide is said to be contra-indicated in the presence of jaundice due to liver metastases (Betteridge, 1964). The presence of liver enlargement due to metastases does not in itself contra-indicate cyclophosphamide therapy, and the author has occasionally noted marked but temporary regression in the size of the liver following continuous low dosage therapy in cases of this type.

### DISCUSSION

The likelihood of a tumour responding to cytotoxic therapy depends not only on the local concentration of the agent, but also on the duration of exposure to the agent. It has been demonstrated for experimental tumours that the response following a given dose of an agent by local arterial infusion, is greater than from the same dose given intravenously. The principle has been confirmed clinically in some tumours, in the increased proportion of responders seen following arterial infusion or perfusion with a cytotoxic agent. The technique is applied more easily in the case of tumours involving the limbs or the head and neck region, but several attempts have been made recently to apply such a technique to breast cancer also (Lentin *et al.*, 1967).

The likelihood of response by a tumour is also influenced by the length of time that it is exposed to an agent, as prolonged exposure is more likely to find all the cells of a slowly dividing tumour in their most sensitive phase. Cyclophosphamide is said to belong to the cycle-specific group of agents which are much more toxic for proliferating cells than for resting cells (Bergsagel, 1969). If this is so, then a short acting agent of this type will require repeated administration in the case of a slowly dividing tumour, to kill the maximum number of malignant cells in their most sensitive phase.

Unfortunately, high local concentration and prolonged exposure are mutually exclusive principles because of the limited tolerance of the host tissues. One must either accept the high single dose, with the aim of high concentration in the tumour, or the lower continuous maintenance dosage with the aim of prolonged exposure time. The relative importance of local concentration and exposure time in each patient for each agent depends on individual recovery factors, and these must be considered separately for the tumour cells as for the host tissue cells.

The relationship between the tissue recovery factors of host and tumour in the cure of experimental tumours is estimated by the therapeutic index. This is defined as the proportion between the smallest lethal dose (LD 5) and the safe curative dose (CD 95). It is therefore an index of therapeutic safety, which has

TABLE II.—*The Therapeutic Index in the Cure of Yoshida Sarcoma in Rats According to the Fractionation of Various Alkylating Agents Given Intravenously* (*after Brock and Wilmanns*, 1958)

| Agent | Daily doses | Therapeutic index |
|---|---|---|
| Cyclophosphamide | 1 | 8·5 |
| | 4 | 4·3 |
| Nitrogen | 1 | 2·2 |
| Mustard oxide | 4 | 2·7 |
| ThioTEPA | 1 | 0·64 |
| | 4 | 0·65 |

been shown to vary considerably between the various alkylating agents according to the fractionation of dosage. Table II shows that in the case of Yoshida sarcoma, fractionation of cyclophosphamide dosage is a disadvantage as shown by a lower therapeutic index. For nitrogen mustard oxide the therapeutic index is slightly better as a result of fractionation of dosage, while for thioTEPA it is unchanged. For obvious reasons we cannot establish such an index in the human, but if the experimental observations could be applied to the human, it might decide the advantage or otherwise of fractionation for curative dosage by each agent.

Clinical observations confirm that the therapeutic index of different agents varies with fractionation. However, we have noted above that in the *palliation* of human mammary cancer by cyclophosphamide, the effect of fractionation is not in agreement with the experimental finding noted in the previous paragraph. It may lead to gross error to carry over into clinical practice, a method of dose fractionation based on the therapeutic index of animal experiment.

The relative importance of dose concentration as against length of exposure to an agent in clinical therapy is also unpredictable on theoretical grounds as the metabolic disposal of the agent will determine its concentration under special fractionation circumstances. The effect of fractionation on each agent therefore needs to be evaluated clinically not only for different tumours, but also for palliative as well as curative purposes. As mentioned above, high dosage therapy may be indicated to achieve dramatic regression of a rapidly growing but localized tumour, because of the high local concentration of the agent over a short period. If the disease is generalized however, the risks outweigh the advantage, and there is no evidence that the likelihood of tumour regression in breast cancer is any greater from intermittent high dosage of cyclophosphamide than from continuous low dose therapy. The mean duration of tumour remission is generally less than 6 months in either case.

" *Is toxicity really necessary?* "

There exists among clinicians practising cytotoxic chemotherapy a widespread belief which is typified by the following opinion—" Some patients will be lucky enough to respond to doses well below the limit of marrow tolerance, but more will respond if the dose is pushed up as nearly as possible to that limit " (Porter, 1962). In a review with this paragraph's title, Bross *et al.* (1966) considered the evidence for the belief that substantial toxicity in the host will be associated with a higher proportion of tumour palliation by cytotoxic agents. They analysed the data from 956 patients receiving 5-fluorouracil, mitomycin C, AB 132, chlorambucil, or 6-mercaptopurine in the treatment of various types of tumour.

Based upon the criterion of 50% reduction in tumour size, Bross found that the proportion of responders was no higher among patients exhibiting signs of marrow toxicity (leucopenia below 2000 per c. mm. or thrombopenia below 100,000 per c. mm.), than among those with evidence of lesser toxicity. A similar observation had been made previously in the specific case of cyclophosphamide therapy in breast or ovarian cancer (Coggins *et al.*, 1961) and of thioTEPA therapy in breast cancer (Lyons and Edelstyn, 1962). The author (Stoll, 1962) had suggested a similar observation in therapy by various alkylating agents in cancers of the breast, lung or ovary.

In cytotoxic therapy aiming at *cure* in the case of *chemoresistant* neoplasms, it is likely that the best results will be achieved when the tumour cells are exposed to the maximum amount of drug which can be tolerated by the marrow. Yet there is no evidence that this likelihood applies to the *palliation* of tumours, particularly *moderately chemosensitive* tumours such as those of the breast, lung and ovary.

This observation can be explained on a theoretical basis by the assumption that even with tumours which are histologically indistinguishable, the chemosensitivity of any particular tumour to a given concentration of a cytotoxic agent is an individual function of the cells of that tumour (Druckrey, 1957). The differing sensitivities shown by individual tumours of the same group will then conform to the basic " law of individual variation " (Fig. 1). This law suggests that the majority of tumours will show average sensitivity, a minority will show marked susceptibility, and a further minority will show marked resistance.

FIG. 1.—Diagram to illustrate law of individual variation as applied to the range of sensitivity to chemotherapy of different members of one tumour type.

If we consider Fig. 1, and assume a tumour type where 50% of the members are known to respond to a specific agent, we note that the chemosensitive members lie to the left of the midline. Those tumours on the extreme left, *i.e.* in the highly susceptible area, will show regression from very low drug concentrations, with almost no associated toxic effects. Those tumours in the middle of the curve will respond to moderate drug concentrations. *If the slope is low* around the middle of the curve, then increase of the dose even to near lethal levels could yield only a very small increase in the proportion of tumour responding, but will lead to a considerable increase in toxicity because of the chemosensitivity of haemopoietic tissue. A small increase in response rate may be undetectable especially if it is mainly at a subclinical level (see introductory section).

The clinician must ask himself before initiating cytotoxic therapy in a patient with cancer whether treatment can possibly cure, or whether it is for palliative purposes. If it is for the latter reason, then the likelihood and degree of possible benefit must outweigh the likelihood and degree of possible morbidity from that specific therapy.

### REFERENCES

ANDERS, C. J.—(1964) Cyclophosphamide Symposium. Editors G. H. Fairley and J. M. Simister. Bristol (Wright) p. 31.
ANDERS, C. J. AND KEMP, N. H.—(1961) *Br. med. J.*, ii, 1516.
BAGSHAWE, K. D.—(1968) *Br. J. Cancer*, **22**, 698.
BERGSAGEL, D. E.—(1969) *Mod. Med. Can.*, **24**, 19.
BETTERIDGE, T. J.—(1964) Cyclophosphamide Symposium. Editors G. H. Fairley and J. M. Simister. Bristol (Wright) p. 25.
BIEDERMANN, C., OBOLENSKY, W., SCOGLIO, P. AND ZURCHER, W. O. (1966) *Cancer Chemother. Abstr.*, **7**, 484.
BOCK, H. E., ALLNER, R. AND GROSS, H. E. (1967). *Cancer Chemother. Abstr.*, **8**, 565.
BROCK, N. AND WILMANNS, H.—(1958) *Germ. med. Mon.*, **3**, 189.
BROSS, I. D. J., RIMM, A. A., SLACK, N. H., AUSMAN, R. K. AND JONES, R.—(1966) *Cancer, N.Y.* **19**, 1780.
BURN, J. I.—(1968) Symp. Cytotoxic Chemotherapy, Forest Med. Soc., p. 8.
CITTADINI, G.—(1966) *Cancer Chemother. Abstr.*, **7**, 365.
COGGINS, P. R., EISMAN, S. H., ELKINS, W. L. AND RAVDIN, R. G.—(1961) *Cancer Chemother. Rep.*, **15**, 3.
CRAN, I.—(1968) Symp. Cytotoxic Chemotherapy, Forest Med. Soc., p. **14**.
DRUCKREY, H.—(1957) *Acta Un. int. Cancr*, **13**, 382.
EDELSTYN, G., GLEADHILL, C. AND LYONS, A.—(1968) *Clin. Radiol.*, **19**, 426.
FORREST, A. P. M.—(1967) *Jl R. Coll. Surg. Edinb.*, **12**, 192.
GEBHARDT, K. H.—(1967) *Cancer Chemother. Abstr.*, **8**, 175.
GERHARTZ, H.—(1964) *Acta Un. int. Cancr.*, **20**, 366.
GORDON, I. AND MCARTHUR, J.—(1965) *Scot. med. J.*, **10**, 27.
HAYAKAWA, T., KANAI, N., YAMADA, R., KURODA, R., HIGASHI, H., MOGAMI, H. AND JINNAI, D.—(1969) *Biochem. Pharmac.*, **18**, 129.
HENNESSY, J. D.—(1966) *Br. med. J.*, ii, 1138.
HURLEY, J. D., TRUMP, D. S., FLATLEY, T. J. AND REISCH, J. D.—(1961) *Archs Surg., Chicago*, **83**, 611.
KENNEDY, B J —(1962) *Ann intern Med.*, **57**, 917.
KOSTANECKI, W., KWIATOWSKA, E. AND ZBORZIC, J.—(1966) *Cancer Chemother. Abstr.*, **7**, 368.
LENTIN, M., NAMBIAR, R. AND BRENNAN, T. G.—(1967) *Br. J. Surg.*, **54**, 519.
LYONS, A. AND EDELSTYN, G.—(1962) *Br. med. J.*, ii, 1280.
OLIVA, L. AND CITTADINI, G.—(1966) *Boll. Soc. ital. Biol. sper.*, **42**, 1107.
PIGATTO, J. C.—(1967) *Cancer Chemother. Abstr.*, **8**, 381.
POMMATAU, B., REVILLARD, J. P. AND GIGNOUX, M.—(1961) *Loire med.*, **65**, 25.
PORTER, E. H.—(1962) *Br. med. J.*, **1**, 869.
DE ROCHEMONT, D. M., RINGLEB, D. AND SCHNEIDER, H.—(1959) *Strahlentherapie*, **109**, 211.
SCHMAHL, D.—(1963) *Cancer Chemother. Abstr.*, **4**, 925.
SHNIDER, B. I.—(1962) *Cancer Chemother. Rep.*, **16**, 61.
SKIPPER, H. E., HEIDELBERGER, C. AND WELCH, A. D.—(1957) *Nature, Lond.*, **179**, 1159.
SNYMAN, H. W.—(1967) *S. Afr. Cancer Bull.*, **11**, 28.
SOUTHAM, C. M.—(1965) *Eur. J. Cancer*, **1**, 173.
STOLL, B. A.—(1962) *Br. med. J.*, ii, **118**, 1128.—(1963) *Br. med. J.*, ii, 210.
STOLL, B. A. AND MATAR, J.—(1961) *Br. med. J.*, ii, 283.
TALLEY, R. W., VAITKEVICIUS, V. K. AND LEIGHTON, G. A.—(1965) *Clin. Pharmac. Ther.*, **6**, 740.